# *CYP3A* genetic variation and taxane-induced peripheral neuropathy: a systematic review, meta-analysis, and candidate gene study

**DOI:** 10.3389/fphar.2023.1178421

**Published:** 2023-07-04

**Authors:** Laurence McEvoy, Joanne Cliff, Daniel F Carr, Andrea Jorgensen, Rosemary Lord, Munir Pirmohamed

**Affiliations:** ^1^ Department of Pharmacology and Therapeutics, University of Liverpool, Liverpool, United Kingdom; ^2^ Clatterbridge Cancer Centre, Liverpool, United Kingdom; ^3^ Health Data Science, University of Liverpool, Liverpool, United Kingdom

**Keywords:** chemotherapy, cytochrome P450, peripheral neuropathy, personalised medicine, pharmacogenetics

## Abstract

**Background:** Taxane-induced peripheral neuropathy (TIPN) is an important cause of premature treatment cessation and dose-limitation in cancer therapy. It also reduces quality of life and survivorship in affected patients. Genetic polymorphisms in the CYP3A family have been investigated but the findings have been inconsistent and contradictory.

**Methods:** A systematic review identified 12 pharmacogenetic studies investigating genetic variation in CYP3A4*22 and CYP3A5*3 and TIPN. In our candidate gene study, 288 eligible participants (211 taxane participants receiving docetaxel or paclitaxel, and 77 control participants receiving oxaliplatin) were successfully genotyped for CYP3A4*22 and CYP3A5*3. Genotyping data was transformed into a combined CYP3A metaboliser phenotype: Poor metabolisers, intermediate metabolisers and extensive metabolisers. Individual genotypes and combined CYP3A metaboliser phenotypes were assessed in relation to neurotoxicity, including by meta-analysis where possible.

**Results:** In the systematic review, no significant association was found between CYP3A5*3 and TIPN in seven studies, with one study reporting a protective association. For CYP3A4*22, one study has reported an association with TIPN, while four other studies failed to show an association. Evaluation of our patient cohort showed that paclitaxel was found to be more neurotoxic than docetaxel (*p* < 0.001). Diabetes was also significantly associated with the development of TIPN. The candidate gene analysis showed no significant association between either SNP (CYP3A5*3 and CYP3A4*22) and the development of TIPN overall, or severe TIPN. Meta-analysis showed no association between these two variants and TIPN. Transformed into combined CYP3A metaboliser phenotypes, 30 taxane recipients were poor metabolisers, 159 were intermediate metabolisers, and 22 were extensive metabolisers. No significant association was observed between metaboliser status and case-control status.

**Summary:** We have shown that the risk of peripheral neuropathy during taxane chemotherapy is greater in patients who have diabetes. CYP3A genotype or phenotype was not identified as a risk factor in either the candidate gene analysis or the systematic review/meta-analysis, although we cannot exclude the possibility of a minor contribution, which would require a larger sample size.

## 1 Introduction

Chemotherapy-induced peripheral neuropathy (CIPN) is a common yet complex adverse effect of some anticancer drugs, and a leading cause of dose reduction and/or premature treatment cessation. Approximately 30%–40% of patients treated with neurotoxic chemotherapeutics will develop CIPN (Staff et al., 2017), although the prevalence ranges from 12%–96% dependent on factors such as regimen and concomitant use of other neurotoxic agents (Pachman et al., 2011; Osmani et al., 2012; Seretny et al., 2014; Eckhoff et al., 2015b; Molassiotis et al., 2019). In cancer survivors, CIPN may persist long-term following treatment, significantly affecting quality of life (Argyriou et al., 2014; Miltenburg and Boogerd, 2014; Cliff et al., 2017). Agents associated with CIPN include taxanes (e.g., docetaxel and paclitaxel) (Velasco and Bruna, 2015), platinum derivative drugs (e.g., carboplatin, cisplatin and oxaliplatin) (Staff et al., 2019), and vinca alkaloids (Grisold et al., 2012).

Taxanes, such as docetaxel and paclitaxel, are commonly used as first-line treatment (monotherapy or in combination) for various solid tumour types including breast, gynaecological, lung, prostate and head-and-neck cancers (Misiukiewicz et al., 2014; Frederiks et al., 2015; Cliff et al., 2017; Ibrahim and Ehrlich, 2020; Velasco-González and Coffeen, 2022). Dose-limiting taxane toxicities include hypersensitivity reactions (Boulanger et al., 2014; Picard and Castells, 2015), gastrointestinal (GI) (Daniels et al., 2008; Jimenez et al., 2011; Liu et al., 2021) and haematological adverse events (Markman, 2003; Frederiks et al., 2015; Tamburin et al., 2019). Around 60%–70% of patients experience taxane-induced peripheral neuropathy (TIPN) (Seretny et al., 2014; Derman and Davis, 2021; Mo et al., 2022), with up to 33% of patients developing severe PN (The National Cancer Institute ‘Common Terminology Criteria Adverse Reactions’ (NCI-CTCAE) grade 3–4) (Lee and Swain, 2006). TIPN is complex and multifactorial, dependent on several risk factors including co-morbidities, chemotherapy regimen, dose-per-cycle, cumulative dose, duration of therapy and concurrent administration of other neurotoxic agents (Lee and Swain, 2006; Osmani et al., 2012; Miltenburg and Boogerd, 2014; Eckhoff et al., 2015b; Tamburin et al., 2019).

TIPN symptoms generally manifest within several weeks of taxane commencement, though both docetaxel and paclitaxel may incite acute neuropathic pain in the first week following the initial dose (Loprinzi et al., 2007; Loprinzi et al., 2011; Reeves et al., 2012; Tanabe et al., 2013; Fernandes et al., 2016; Starobova and Vetter, 2017). Classically, TIPN manifests in a ‘glove-and-stocking’ distribution, starting in the fingers and toes and may progress to the hand/wrist and lower leg. Sensory symptoms are most common and include numbness and tingling, dysaesthesia and paraesthesia, and may progress to painful and burning sensations. Motor symptoms are less common, manifesting as muscle weakness, and impaired fine motor movement, and are usually associated with higher doses (Chaudhry et al., 1994; Freilich et al., 1996). Motor impairment can result in significant loss of functional abilities including balance (Tofthagen et al., 2013; Miltenburg and Boogerd, 2014; Ibrahim and Ehrlich, 2020), with potential for mobility-related disability (Hile et al., 2010). Cranial nerve involvement has been reported anecdotally (Velasco and Bruna, 2015). Uncommon autonomic symptoms include digestive, sexual, and urinary disruption (Mols et al., 2016; Zajaczkowska et al., 2019). Development of TIPN is an indication for dose reduction or discontinuation, both resulting in sub-optimal chemotherapy regimens with potential to affect cancer treatment outcomes and overall survival.

Despite recent developments in identifying predictive and blood biomarkers of PN using ultrasensitive protein assays (Rossor and Reilly, 2022), there are currently no universally-accepted gold standard diagnostic or assessment tools for CIPN (McCrary et al., 2017; Ibrahim and Ehrlich, 2020). The NCI-CTCAE is the most commonly used assessment tool in clinical practice (Tan et al., 2019; Li et al., 2020; Selvy et al., 2021), yet a 2017 systematic review identified 117 distinct CIPN assessment tools (McCrary et al., 2017).

Diagnosis, assessment, and management of CIPN is challenging. Key patient characteristics increasing the risk of CIPN (including PN severity and long-duration PN), have been identified (summarised in Table 1), including obesity (Bao et al., 2016; Bandos et al., 2018)/high body mass index (BMI) (Bao et al., 2016; Hiramoto et al., 2022), and advanced age (Tanabe et al., 2013; Schneider et al., 2015; Bao et al., 2016; Hershman et al., 2016; Tanabe et al., 2017; Bandos et al., 2018; Miaskowski et al., 2018; Molassiotis et al., 2019; Sánchez-Barroso et al., 2019; Hiramoto et al., 2022; Rattanakrong et al., 2022), although age has been contested as an independent risk factor (Eckhoff et al., 2015b; Johnson et al., 2015; Barginear et al., 2019; Sánchez-Barroso et al., 2019; Valentine, 2020). Multimorbidity/co-morbidity (Loprinzi et al., 2007; Lavoie Smith et al., 2011; Reeves et al., 2012; Tanabe et al., 2013; de la Morena Barrio et al., 2015; Bao et al., 2016; Fernandes et al., 2016; Hershman et al., 2016; Molassiotis et al., 2019) is a further risk factor. A predisposition to CIPN has been reported in individuals suffering from nerve damage due to alcohol, inherited neuropathy and most notably, diabetes (Quasthoff and Hartung, 2002; de la Morena Barrio et al., 2015; Hershman et al., 2016; Ottaiano et al., 2016; Molassiotis et al., 2019). Peripheral neuropathy is a common manifestation of both type 1 and type 2 diabetes (diabetic peripheral neuropathy) (Selvarajah et al., 2019), and patients with pre-existing diabetes mellitus tend to experience more dose delays and dose reductions, often with long-lasting, significant CIPN (de la Morena Barrio et al., 2015). Evidence suggests severe PN is an independent risk factor associated with persisting PN (Tanabe et al., 2013). Interestingly, recent evidence suggests paclitaxel-induced PN may also be influenced by the microbiome (Castelli et al., 2018; Was et al., 2022). Other risk factors include polypharmacy (Sánchez-Barroso et al., 2019), index drug (Argyriou et al., 2014), the number of chemotherapy cycles (Molassiotis et al., 2019), and total cumulative dose (Argyriou et al., 2008; Seretny et al., 2014; Hiramoto et al., 2022).

**TABLE 1:** Reported risk factors for developing chemotherapy-induced peripheral neuropathy.

Patient characteristic	Description	References
Patient factors		
Advanced Age	>60 years; >65 years	Molassiotis et al. (2019), Miaskowski et al. (2018), Rattanakrong et al. (2022), Schneider et al. (2015), Sánchez-Barroso et al. (2019), Hershman et al. (2016), Bandos et al. (2018), Bao et al. (2016), Hiramoto et al. (2022), Tanabe et al. (2013), Tanabe et al. (2017), Rattanakrong et al. (2022), Bao et al. (2016)
Race	African Americans	Simon et al. (2017), Schneider et al. (2012), Schneider et al. (2015), Bhatnagar et al. (2014)
Co-morbidity/multimorbidity	Diabetes mellitus; Taxane-induced pain syndrome (TAPS); Peripheral nervous system disorders; Psychological: anxiety, depression, insomnia	Molassiotis et al. (2019), Lavoie Smith et al. (2011), Molassiotis et al. (2019), Hershman et al. (2016), de la Morena Barrio et al. (2015) Loprinzi et al. (2007), Reeves et al. (2012), Tanabe et al. (2013), Fernandes et al. (2016), Hausheer et al. (2006), Bao et al. (2016)
Clinical history	Previous neuropathy; (Chronic) increased alcohol consumption; Obesity; Higher body mass index	Molassiotis et al. (2019), Bandos et al. (2018), Molassiotis et al. (2019), Bandos et al. (2018), Bao et al. (2016), Bao et al. (2016), Hiramoto et al. (2022)
Drug factors		
Chemotherapy regimen	Drug; Number of chemotherapy cycles; Cumulative dose	Argyriou et al. (2014), Molassiotis et al. (2019), Seretny et al. (2014), Argyriou et al. (2008), Hiramoto et al. (2022)
Polypharmacy		Sánchez-Barroso et al. (2019)
Concomitant drug use	Cardiovascular drugs; Other neurotoxic drugs	Sánchez-Barroso et al. (2019), Valentine (2020), Johnson et al. (2015)

Genetic factors predisposing to TIPN have been investigated, yet findings have been inconsistent and contradictory. To date, most investigations have been candidate gene studies (Leskelä et al., 2011; Bergmann et al., 2012; de Graan et al., 2013; Bosó et al., 2014; Eckhoff et al., 2015a; Apellaniz-Ruiz et al., 2015; Lambrechts et al., 2015; Hu et al., 2016; Di Francia et al., 2017; Ciruelos et al., 2019), as opposed to genome-wide association studies (Schneider et al., 2015) or whole-exome sequencing (WES) analysis (Shen et al., 2023). A high degree of heterogeneity has been reported in taxane pharmacokinetics (PK) (Michael et al., 2011; Jabir et al., 2012; Hertz, 2013; Bosó et al., 2014; Sim et al., 2018). Docetaxel is largely metabolized by the cytochrome P450 3A (CYP3A) enzymes CYP3A4 and CYP3A5 (Shou et al., 1998; Engels et al., 2004; Powell et al., 2021), while paclitaxel is metabolized by CYP3A4 and CYP2C8 (Hertz, 2013; Wang et al., 2014; Marcath et al., 2019). Single nucleotide polymorphisms in genes encoding these CYP3A enzymes are known to affect their function, with reduced or loss of function variants often associated with increased toxicities. The CYP3A4*22 variant (allele frequency of 5%–7% in Caucasian populations) is associated with decreased CYP3A4 activity (Elens et al., 2011a; Elens et al., 2011b; Elens et al., 2011c; Elens et al., 2012; Elens et al., 2013a; Elens et al., 2013b; van der Weide and van der Weide, 2014; de Jonge et al., 2015; Sanchez Spitman et al., 2017; Mulder et al., 2021). In CYP3A5, the functional CYP3A5*1 allele is present in approximately 7% of Caucasians. The loss of function variant, CYP3A5*3, results in the production of non-functional proteins (Sanchez Spitman et al., 2017; Scheibner et al., 2018).

The primary aims of our study were to i) perform a systematic review, evaluating the current literature for potential CYP3A4*22 and CYP3A5*3 involvement as genetic risk factors for TIPN and ii) using a candidate genotype approach, interrogate potential associations between these pharmacokinetic genetic variants, CYP3A4*22 and CYP3A5*3, and susceptibility to TIPN, evaluating the combined influence of these 2 SNPs as a genotype-derived combined CYP3A metaboliser phenotype as a genetic risk factor for TIPN.

## 2 Materials and methods

### 2.1 Systematic review

#### 2.1.1 Search strategy

PubMed, Science Direct and the Cochrane Library were searched on 23/11/2022, applying the search strategy shown in Supplementary Table S1, based on the PRISMA guidelines (PRISMA, Preferred Reporting Items for Systematic Reviews and Meta-Analyses) (Page et al., 2021). Searches were not limited by date restrictions. During the review process of our manuscript, a further study was identified (published May 2023) and subsequently included in the systematic review prior to final submission.

#### 2.1.2 Study selection and review

**FIGURE 1:**
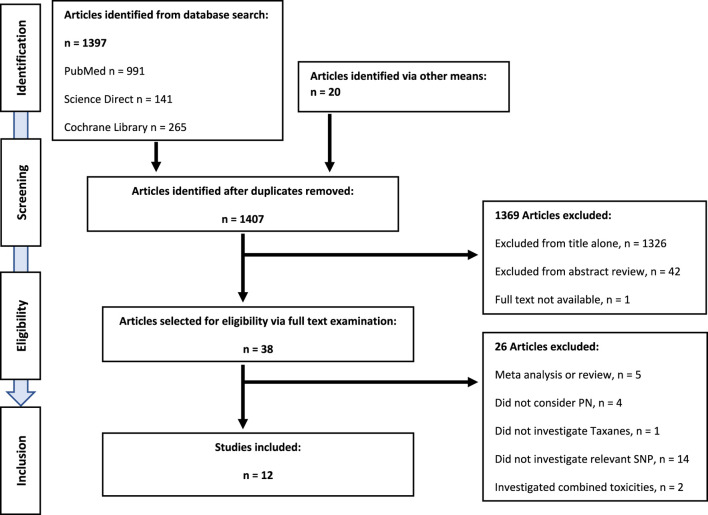
PRISMA diagram showing systematic literature review, and identification of eligible publications.

English language articles investigating an association between single nucleotide polymorphisms in CYP3A4*22 and CYP3A5*3 and peripheral neuropathy outcomes in adult populations receiving anti-cancer taxane chemotherapy regimens were included. Search results were identified, compiled, and screened (LMc). Irrelevant articles were excluded in the first instance by title, then by abstract. Full text examination of remaining articles assessed eligibility and identified manuscripts for inclusion (LMc). Reference lists from reviews and eligible studies were also screened for suitable articles. The article identification and selection process is outlined in the schematic (Figure 1).

The following relevant clinical and methodological information was extracted from the manuscripts: Allele, chemotherapy (treatment), phenotype definition, ethnicity, participant information (sample size and cancer type) and main findings. The data is presented in Table 2.

**TABLE 2:** Study characteristics and principal findings from the systematic review.

References	Chemotherapy	PN phenotype definition	Ethnicity	Participants	Main findings	Inclusion in meta-analysis
CYP3A4*22						
Apellaniz-Ruiz et al. (2015)	Paclitaxel	NCI-CTCAE v4.0	Spanish	Country: Spain. Total: 236; Breast and ovarian cancer patients	Trend toward higher treatment modifications in carriers of CYP3A4*22 (*p* = 0.066); yet no statistically significant differences observed for PN grade and treatment modifications due to PN	Insufficient data available for inclusion in meta-analysis
Ciruelos et al. (2019)	Paclitaxel (nab-paclitaxel)	NCI-CTCAE v4.0	Spanish	Country: Spain. Total: 60; Breast cancer patients	No correlation between CYP3A4*22 and neurotoxicity in either univariate (*p* = 0.562, HR = 1.43, 95% CI = 0.43–4.79) or multivariate analysis. (*p* = 0.241, HR = 2.12, 95% CI = 0.60–7.49)	Insufficient data available for inclusion in meta-analysis
de Graan et al. (2013)	Paclitaxel	NCI-CTCAE v2.0–4.0	Exploratory cohort: Caucasian, 96%; Other, 4%	Country: Netherlands. Exploratory cohort: 261, various cancer types	Female CYP3A4*22 carriers have increased risk of PN, *p* = 0.043	Included in meta-analysis. Results displayed in Forest Plot, Figure 2
			Validation cohort: Caucasian, 95%; Other, 3%; Unknown, 2%	Validation cohort: 239, various cancer types	CYP3A4*22 carriers have increased risk of grade 3 PN (*p* = 0.001, OR = 19.1, 95% CI = 3.3–110), confirming observations from the exploratory cohort in females	
Di Francia et al. (2017)	Taxane: Paclitaxel- or Docetaxel-based	NCI-CTCAE v4.0	Italian, Caucasian	Country: Italy. 76 cancer patients; various cancer types. 35 receiving adjuvant taxane chemotherapy. Control cohort, n = 41; Case (taxane) cohort = 35	Pharmacogenomic analysis showed no correlation between CYP3A4*22 and neurotoxicity	Included in meta-analysis. Results displayed in Forest Plot, Figure 2
Shen et al. (2023)	Paclitaxel (adjuvant)	NCI-CTCAE v3.0	European ancestry	Country: United States of America (patients genetically determined to be of European ancestry). 340 breast cancer patients (168 cases, PN grade 3–4; 172 controls)	No association observed between *CYP3A4* metaboliser status and severe TIPN	Insufficient data available for inclusion in meta-analysis. Allele frequencies reported for the total study population, rather than by cases and tolerant controls
CYP3A5*3						
Bergmann et al. (2012)	Paclitaxel (followed by carboplatin)	NCI-CTCAE v3.0	Scandinavian, Caucasian	Country: Denmark/Sweden. 92; Ovarian, fallopian tube or peritoneal cancer patients	No association between CYP3A5*3 variant and PN reported	Insufficient data available for inclusion in meta-analysis
Bosó et al. (2014)	Docetaxel, n = 70. Paclitaxel, n = 43	NCI-CTCAE v4.0	Caucasian	Country: Spain. 113 breast cancer patients	No association between CYP3A5*3 variant and PN reported	Insufficient data available for inclusion in meta-analysis
Eckhoff et al. (2015a)	Docetaxel	NCI-CTCAE v2.0	Western European	Country: Denmark. 150 (early-stage) breast cancer patients	No association between CYP3A5*3 variant and PN reported	Included in meta-analysis. Results displayed in Forest Plot, Figure 2
Hu et al. (2016)	Paclitaxel/carboplatin regimen	WHO grading scale/NCI-CTCAE v2.0	Chinese	Country: China. 75 epithelial ovarian cancer patients	No association observed between CYP3A5*3 variant and PN	Included in meta-analysis. Results displayed in Forest Plot, Figure 2
Lambrechts et al. (2015)	Paclitaxel (Paclitaxel-carboplatin combination therapy)	NCI-CTCAE v4.0	Caucasian, 99%	Country: Belgium/Luxembourg. 265 ovarian cancer patients receiving paclitaxel-carboplatin combination therapy underwent neurotoxicity analysis	No significant association between CYP3A5*3 variant and PN	Insufficient data available for inclusion in meta-analysis
Leskelä et al. (2011)	Paclitaxel	NCI-CTCAE v2.0	White, and of European origin	Country: Spain. 118 cancer patients; various cancer types	CYP3A5*3 variant associated with decreased risk of PN (*p* = 0.012, HR = 0.51, 95% CI = 0.30–0.86; HR estimated by multivariable Cox regression, adjusting for treatment schedule and age)	Insufficient data available for inclusion in meta-analysis
Schneider et al. (2015)	ECOG-5103: Paclitaxel	NCI-CTCAE v3.0	White, African American; European descent subset	Country: United States. ECOG-5103: 3,431 Breast cancer patients	GWAS: No association between CYP3A5*3 variant and PN reported	Insufficient data available for inclusion in meta-analysis
	ECOG-1199 (validation study): Paclitaxel or docetaxel	NCI-CTCAE v2.0	White, African American; European descent (Caucasian) subset	ECOG-1199: 2,407 Breast cancer patients	No association between CYP3A5*3 variant and PN reported	Insufficient data available for inclusion in meta-analysis
Shen et al. (2023)	Paclitaxel (adjuvant)	NCI-CTCAE v3.0	European ancestry	Country: United States of America (patients genetically determined to be of European ancestry). 340 breast cancer patients (168 cases, PN grade 3–4; 172 controls)	No association observed between *CYP3A5* metaboliser status and severe TIPN	Insufficient data available for inclusion in meta-analysis. Allele frequencies reported for the total study population, rather than by cases and tolerant controls

ABBREVIATIONS: CI, confidence interval; HR, hazard ratio; OR, odds ratio; PN, peripheral neuropathy; WHO, world health organization.

### 2.2 Candidate gene study

#### 2.2.1 Participants, recruitment and consent

Participants were recruited retrospectively using a multi-centre ethics approved protocol, *Molecular Genetics of Adverse Drug Reaction* (NIHR Portfolio Study ID 8630). All patients were given a Patient Information Leaflet (PIL) and a signed consent form was required for recruitment and to be included in the study. If a patient lacked capacity to consent, a personal consultee or a nominated consultee was approached. Recruitment took place across five sites located throughout England: Clatterbridge Cancer Centre NHS Foundation Trust (CCO), St Helens and Knowsley NHS Trust (SHK), The Christie NHS Foundation Trust (CHR), Wrightington, Wigan and Leigh NHS Foundation Trust (WWL), and The Shrewsbury and Telford Hospital NHS Trust (RSO).

Eligible patients, aged >18 years and meeting the case or control definitions, were invited to participate in the study. Identification of eligible patients was *via* the following methods: patient lists, generated by coding departments, of individuals having received the relevant chemotherapy regimens; patient consultants informing the trials/study team; and identification on chemotherapy day units. Participants receiving oxaliplatin represented a control cohort in this study. The following were the criteria for categorisation as either case or control:

**Case** definition:

• No pre-existing peripheral neuropathy at the time of starting chemotherapy.
• Grade 2 or greater peripheral neuropathy leading to dose reduction, dose delay, or early cessation of chemotherapy.

OR.

Any grade 3 or 4 peripheral neuropathy developing during or within 6 weeks of completion of chemotherapy (this was also defined as a severe case).

• Chemotherapy regimens included were oxaliplatin, 3 weekly paclitaxel, and any three weekly docetaxel regimen.
• No subsequent treatment with cisplatin or vinca alkaloid.

**Control** definition:

• Has received at least 6 cycles of oxaliplatin containing chemotherapy OR 6 cycles of 3- weekly paclitaxel chemotherapy, OR 6 or more cycles of docetaxel 75 mg/m^2^ OR 4 or more cycles of docetaxel 100 mg/m^2^.
• No pre-existing peripheral neuropathy at the time of starting chemotherapy.
• No peripheral neuropathy during chemotherapy OR maximum of grade 1 peripheral neuropathy.
• No subsequent treatment with cisplatin or a vinca alkaloid.

Peripheral neuropathy grading was confirmed using the National Cancer Institute Common Terminology Criteria for Adverse Events NCI-CTCAE v4.0 (https://ctep.cancer.gov/protocolDevelopment/electronic_applications/ctc.htm) (National Cancer Institute, 2010). Maximal symptoms and symptoms at recruitment, including trips or falls due to PN, were recorded. Information on family history of PN and pre-existing PN was also captured.

Treatment information details were obtained from patient case notes, including chemotherapy regimen, cumulative dose of index drug, oncology treatment medications and alterations to planned chemotherapy regimen (if any): dose delays, reductions, and premature cessation of treatment. ADR data was recorded: date of onset of reaction, resolution of ADR, and if resolved, date thereof. Concurrent medications/chemicals (at time of reaction) were recorded. All case report form (CRF) data was transcribed to ‘OpenClinica’ eCRFs (electronic CRFs) facilitating clinical data management (OpenClinica LLC, Waltham, MA, USA).

#### 2.2.2 Sample collection

Approximately 9 mL whole blood was collected in BD Vacutainer^®^ EDTA (BD™ Biosciences, USA) or S-Monovette^®^ EDTA tubes (Sarstedt AG & Co. KG, Germany). If venepuncture was unsuccessful or not possible, 2–4 mL saliva was collected using Norgen^©^ Saliva DNA Collection and Preservation Devices (Product #: RU49000; Norgen Biomek Corp., Ontario, Canada) or DNA Genotek Oragene^®^ DNA OG-500 kits (DNA Genotek Inc., Ontario, Canada). Blood samples were stored at −20°C prior to DNA isolation. Saliva samples were stored at 4°C prior to DNA isolation.

#### 2.2.3 DNA isolation and quantification

Genomic DNA was isolated from 5 mL whole blood using the Chemagic™ Magnetic Separation Module 1 (MSM 1 (PerkinElmer^®^, USA)) as per manufacturer’s protocol (Chemagen Biopolymer-Technologie AG, Baesweiler, Germany) using CMG-703–1 Chemagic DNA Blood Kits (CMG-703–1 Chemagic DNA Blood 5k Kit H12, PerkinElmer LAS (UK) Ltd, Buckinghamshire, UK). Genomic DNA was manually isolated from 2–4 mL saliva using the Norgen^©^ Saliva DNA Isolation Reagent Kit (Product #: RU35720; Norgen Biomek Corp., Ontario, Canada) or Oragene prepIT^®^ L2P DNA extraction kit (DNA Genotek Inc., Ontario, Canada), as per manufacturer’s protocols. Quantification of DNA samples was initially completed using the NanoDrop™ 8,000 Spectrophotometer (ThermoFisher Scientific™, USA). DNA concentration was confirmed using the Quant-iT™ PicoGreen™ dsDNA quantitation assay kit (ThermoFisher Scientific™, USA).

#### 2.2.4 Genotyping by TaqMan^®^ real-time polymerase chain reaction (qPCR)

Genotyping of CYP3A4*22 and CYP3A5*3 was undertaken using commercially available, validated Taqman™ drug metabolism SNP genotyping assays (CYP3A4*22, NCBI SNP Reference: rs35599367, Assay ID: C_59013445_10; CYP3A5*3, NCBI SNP Reference: rs776746, Assay ID: C_26201809_30) with 1 x Genotyping Master Mix (all acquired from Applied Biosystems (ThermoFisher Scientific™, USA)), using the Applied Biosystems 7900HT Fast Real-Time PCR System (Applied Biosystems, ThermoFisher Carlsbad, CA), with Sequence Detection Systems (SDS) Version 2.4.1 software (2010 Life Technologies Corp.). 5 μL reaction volumes were used. All samples were analysed in duplicate. SNPs with a call rate of <95% were excluded. Minor allele frequencies (MAF) for both SNPs were confirmed using dbSNP: CYP3A4*22 MAF European (Caucasian) populations, 5%. CYP3A5*3 MAF European (Caucasian) populations, 7%.

#### 2.2.5 Statistics and data analysis

Statistical analysis was undertaken using the SPSS^®^ statistical package (IBM Corp. Released 2021. IBM SPSS Statistics for Windows, Version 28.0. Armonk, NY: IBM Corp). Continuous variables were analysed by student’s (independent) *t*-test, and categorical variables by the chi-square test.

Genotype-derived CYP3A (*CYP3A4/CYP3A5*) metaboliser phenotypes were defined as previously described (Sim et al., 2018), as follows (Supplementary Table S2):

• Poor metabolisers (PM); expected poor catabolic activity, carrying 1 or 0 active *CYP3A4* or *CYP3A5* alleles.
• Intermediate metabolisers (IM); expected intermediate catabolic activity, carrying 2 active *CYP3A4* and *CYP3A5* alleles.
• Extensive metabolisers (EM); expected extensive catabolic activity, carrying at least 3 active *CYP3A4* and *CYP3A5* alleles.

Univariate analysis analysed SNP association between case, severe case, and control groups. Clinical factors with significant differences (*p* = <0.1) were carried forward for inclusion in the binary logistic regression model.

#### 2.2.6 Meta-analysis

Forest plots were produced using RevMan (Review Manager (RevMan) [Computer program]. Version 5.4., 2020), as described in Table 2. The Forest Plots were inspected and assessed for statistical heterogeneity; categorization of heterogeneity was based on the Cochrane Handbook for Systematic Reviews of Interventions: 0%–40% might not be important; 30%–60% may represent moderate heterogeneity; 50%–90% may represent substantial heterogeneity; 75%–100% considerable heterogeneity.

## 3 Results

### 3.1 Systematic review

Twelve studies met the predefined eligibility criteria and were included in the systematic review (Figure 1). There was a single genome-wide association study (GWAS), with a validation cohort including multiple candidate SNPs across multiple genes (Schneider et al., 2015). Three studies investigated SNPs in a single candidate gene (de Graan et al., 2013; Apellaniz-Ruiz et al., 2015; Hu et al., 2016). Seven studies investigated multiple SNPs across multiple genes (Leskelä et al., 2011; Bergmann et al., 2012; Bosó et al., 2014; Eckhoff et al., 2015a; Lambrechts et al., 2015; Di Francia et al., 2017; Ciruelos et al., 2019). The remaining study employed genome-wide genotyping, whole-exome sequencing (WES) analyses, and TaqMan assays (Shen et al., 2023). Assessing the association between CYP3A4*22 and TIPN were four studies (de Graan et al., 2013; Apellaniz-Ruiz et al., 2015; Di Francia et al., 2017; Ciruelos et al., 2019). Seven studies assessed the association between CYP3A5*3 and TIPN (Leskelä et al., 2011; Bergmann et al., 2012; Hertz et al., 2012; Bosó et al., 2014; Lambrechts et al., 2015; Schneider et al., 2015; Hu et al., 2016). A single study assessed the association between both SNPs, CYP3A4*22 and CYP3A5*3, and TIPN (Shen et al., 2023).

All studies were published between 2011 and 2023. Across all 12 studies, assessment of PN was made using NCI-CTCAE, although the version used differed between studies as shown in Table 2. In addition to evaluation of toxic effects using NCI-CTCAE, one study also used the World Health Organization grading scale to assess neurotoxicity (Hu et al., 2016). Nine studies were conducted in Europe. These studies reported ethnicity information as European or Caucasian (>95%) as described in Table 2. One study was conducted in China (Hu et al., 2016), with exclusively Chinese participants, and two in the United States (Schneider et al., 2015; Shen et al., 2023): one of these US studies analysed patients of European ancestry (Shen et al., 2023). Seven studies focussed on single cancer types: five studies on breast cancer (Bosó et al., 2014; Eckhoff et al., 2015a; Schneider et al., 2015; Ciruelos et al., 2019; Shen et al., 2023), and two on ovarian cancer (Lambrechts et al., 2015; Hu et al., 2016). One study recruited both breast and ovarian cancer patients (Apellaniz-Ruiz et al., 2015), and another recruited gynaecological cancers (ovarian, fallopian tube and peritoneal cancers) (Bergmann et al., 2012). The remaining studies included participants with various cancer types (Leskelä et al., 2011; de Graan et al., 2013; Di Francia et al., 2017). Considering chemotherapy regimens, eight studies were taxane-only therapies (Leskelä et al., 2011; de Graan et al., 2013; Bosó et al., 2014; Eckhoff et al., 2015a; Apellaniz-Ruiz et al., 2015; Schneider et al., 2015; Di Francia et al., 2017; Ciruelos et al., 2019). Three studies recruited patients treated with paclitaxel plus carboplatin combination therapy (Bergmann et al., 2012; Lambrechts et al., 2015; Hu et al., 2016). The final study recruited patients treated with adjuvant paclitaxel (Shen et al., 2023).

### 3.2 Findings of the systematic review

For CYP3A4*22 ***,*** there were contradictory findings in the literature. Di Francia *et al* showed no correlation between CYP3A4*22 and neurotoxicity after treatment with docetaxel or paclitaxel (Di Francia et al., 2017). Ciruelos *et al* also showed no significant association between CYP3A4*22 and neurotoxicity after treatment with nab-paclitaxel (Ciruelos et al., 2019). Conversely, a Dutch trial reported a significant association between female CYP3A4*22 carriers and paclitaxel-induced neurotoxicity in an exploratory cohort (*p* = 0.043). An independent validation cohort reported a higher risk of developing grade 3 neurotoxicity in CYP3A4*22 carriers compared to non-carriers (*p* = 0.001; odds ratio (OR) = 19.1, 95% confidence interval (CI) = 3.3–110) (de Graan et al., 2013). Whole-exome sequencing of gene *CYP3A4* in Spanish participants receiving paclitaxel chemotherapy reported a trend towards higher treatment modifications in carriers of CYP3A4*22 (*p* = 0.066), but no statistically significant differences were observed for neuropathy grade and treatment modifications due to neuropathy (Apellaniz-Ruiz et al., 2015).

For CYP3A5*3, out of the seven studies, no significant association between the CYP3A5*3 variant and PN was reported in six (Bergmann et al., 2012; Bosó et al., 2014; Eckhoff et al., 2015a; Lambrechts et al., 2015; Schneider et al., 2015; Hu et al., 2016). The remaining study (Leskelä et al., 2011), in 118 Spanish paclitaxel-treated cancer patients reported a protective association for CYP3A5*3 (*p* = 0.012, hazard ratio per allele = 0.51, 95% CI = 0.30–0.86).

Shen *et al* analysed both CYP3A4*22 and CYP3A5*3 in breast cancer patients of European ancestry receiving standard doses of paclitaxel*.* Four diplotypes for *CYP3A4* (*1/*1, *1/*2, *1/*22 and *22/*22) and three for *CYP3A5* (*1/*1, *1/*3, and *3/*3) were identified. Metaboliser status of *CYP3A4*, *CYP3A5* (and *CYP2C8*) was predicted for each participant. No associations were reported between *CYP3A4* or *CYP3A5* predicted metaboliser status and severe (grade 3–4) TIPN (Shen et al., 2023).

### 3.3 Candidate gene study

Twenty-four participants were excluded due to genotyping failures despite repeat genotyping. A total of 288 eligible participants with full clinical data were successfully genotyped and included in the final analysis. All participants were Caucasian.

Of the total study population (*n* = 288), 211 (73%) were receiving taxane chemotherapy (docetaxel or paclitaxel): of these, 54 (26%) were categorised as cases, with 27 (13%) having severe neuropathy. Therefore, 157 (74%) participants served as controls (Table 3).

**TABLE 3:** Case and control comparison of non-genetic clinical variables in the taxane cohort.

Variable		Population (n = 211)	Controls (n = 157)	Peripheral neuropathy (n = 54)	*p*-value	Severe peripheral neuropathy (n = 27)	*p*-value
Index Drug	Docetaxel	139 (66%)	114 (73%)	25 (46%)	<0.001	12 (44%)	0.006
	Paclitaxel	72 (34%)	43 (27%)	29 (54%)		15 (56%)	
Mean Age, Years (SD)		60.6 (±11.3)	59.7 (±11.6)	63.0 (±10.2)	0.065	63.3 (±9.4)	0.133
Sex	Male	55 (26%)	50 (32%)	5 (9%)	0.001	4 (15%)	0.107
	Female	156 (74%)	107 (68%)	49 (91%)		23 (85%)	
Mean BMI (SD)		28.2 (±5.6)	27.8 (±5.3)	29.4 (±6.3)	0.103	29.3 (±5.9)	0.196
Diabetes	No	198 (94%)	151 (96%)	47 (87%)	0.024	22 (81%)	0.012
	Yes	13 (6%)	6 (4%)	7 (13%)		5 (19%)	
Alcohol Consumption, units/day	< 1	140 (66%)	103 (66%)	37 (69%)	0.846	17 (63%)	0.923
	1–5	54 (26%)	41 (26%)	13 (24%)		8 (30%)	
	6–14	15 (7%)	11 (7%)	4 (7%)		2 (7%)	
	15 +	2 (1%)	2 (1%)	0 (0%)		0 (0%)	
Hepatic Impairment	No	211 (100%)	157 (100%)	54 (100%)	-	27 (100%)	-
CYP3A-interacting concurrent medications	Yes	211 (100%)	157 (100%)	54 (100%)	-	27 (100%)	-

These bold values are simply sub-categories for each variable.

A cohort of 77 (27%) patients who had received oxaliplatin chemotherapy was also studied. This included patients with (*n* = 38; 49%; 20 (26%) were severe) and without (*n* = 39; 51%) peripheral neuropathy.

For the taxane cohort, demographics and non-genetic patient characteristics are shown in Table 3. The most common primary cancer types were breast (*n* = 81 (38%)), followed by ovarian (*n* = 57, 27%) and prostate (*n* = 53; 25%). The mean age of the taxane cohort was 60.6 years (standard deviation (SD) ±11.3). Mean age for control participants was 59.7 years (standard deviation (SD) ±11.6).

Paclitaxel was significantly more neurotoxic than docetaxel (*p* ≤ 0.001; chi-square). Diabetes was also found to be significantly associated with neuropathy: 4% of control patients had diabetes, whilst 13% of case patients had diabetes (*p* = 0.024). In severe PN cases, 19% of patients had diabetes (*p* = 0.012). No significant differences were observed between cases and controls in BMI, alcohol consumption (units/day), hepatic impairment or CYP3A-interacting concurrent medications. Cases tended to be older although this was not significant (*p* = 0.065).

For the taxane cohort, both SNPs were confirmed to be in Hardy-Weinberg equilibrium (CYP3A4*22, *p* = 0.25; CYP3A5*3, p = 0.40). No significant associations were observed between either SNP and development of peripheral neuropathy or severe peripheral neuropathy (Table 4). Combined *CYP3A4* and *CYP3A5* genotypes were transformed into combined CYP3A metaboliser phenotype classifications (Sim et al., 2018), as shown in Supplementary Tables S2 and S5. No significant associations were observed between metaboliser phenotype and case-control status *or* severe case-control status (Tables 4 and 5). No significant association was observed between either SNP and the development of peripheral neuropathy in the taxane-treated participants in univariate or multivariate analysis after adjustment for relevant clinical factors.

**TABLE 4:** CYP3A genotypes in the taxane and oxaliplatin cohorts with and without peripheral neuropathy.

Gene	SNP	Genotype	Control	PN	Or (95% CI)	*p*-value	Severe PN	Or (95% CI)	*p*-value
Taxane cohort									
CYP3A4*22	*rs35599367*	*1/*1	132 (84%)	48 (89%)	0.6 (0.2–1.6)	0.3	24 (89%)	0.6 (0.2–2.4)	0.5
		*1/*22	25 (16%)	6 (11%)			3 (11%)		
		*22/*22	0	0			0		
CYP3A5*3	*rs776746*	*1/*1	0	0	1.2 (0.4–3.8)	0.7	0	1.4 (0.4–5.7)	0.6
		*1/*3	18 (11%)	5 (9%)			3 (11%)		
		*3/*3	139 (89%)	49 (91%)			24 (89%)		
Oxaliplatin cohort									
CYP3A4*22	*rs35599367*	*1/*1	37 (95%)	37 (97%)		1.0	20 (100%)		0.5
		*1/*22	2 (5%)	1 (3%)			0		
		*22/*22	0	0			0		
CYP3A5*3	*rs776746*	*1/*1	0	0		0.1	0		1.0
		*1/*3	15 (38%)	8 (21%)			7 (35%)		
		*3/*3	24 (62%)	30 (79%)			13 (65%)		

CI, confidence interval; OR, odds ratio; PN, peripheral neuropathy.

These rs numbers are the reference SNP values for identifying the particular SNPs.

**TABLE 5:** CYP3A metaboliser status for the taxane and oxaliplatin cohorts.

Metaboliser status	Controls	Cases with peripheral neuropathy	Cases with severe peripheral neuropathy
Taxane cohort (numbers)			
Poor Metabolizer	24	6	3
Intermediate Metabolizer	116	43	21
Extensive Metabolizer	17	5	3
OR (95% CI)		1.5 (0.7–3.0)	1.5 (0.6–3.7)
*p*-value		0.3	0.4
Oxaliplatin cohort (numbers)			
Poor Metabolizer	2	1	0
Intermediate Metabolizer	22	29	13
Extensive Metabolizer	15	8	7
*p*-value		0.2	0.5

CI, confidence interval; OR, odds ratio.

For the oxaliplatin cohort, both SNPs were confirmed to be in Hardy-Weinberg equilibrium (CYP3A4*22, *p* = 0.86; CYP3A5*3, p = 0.12). As expected, no significant association was observed between either SNP and development of peripheral neuropathy or severe peripheral neuropathy (Table 4). No significant associations were observed between genotype-derived combined CYP3A metaboliser phenotype (*CYP3A4*/*CYP3A5*) and development of peripheral neuropathy or severe peripheral neuropathy (Table 5).

### 3.4 Meta-analysis

**FIGURE 2:**
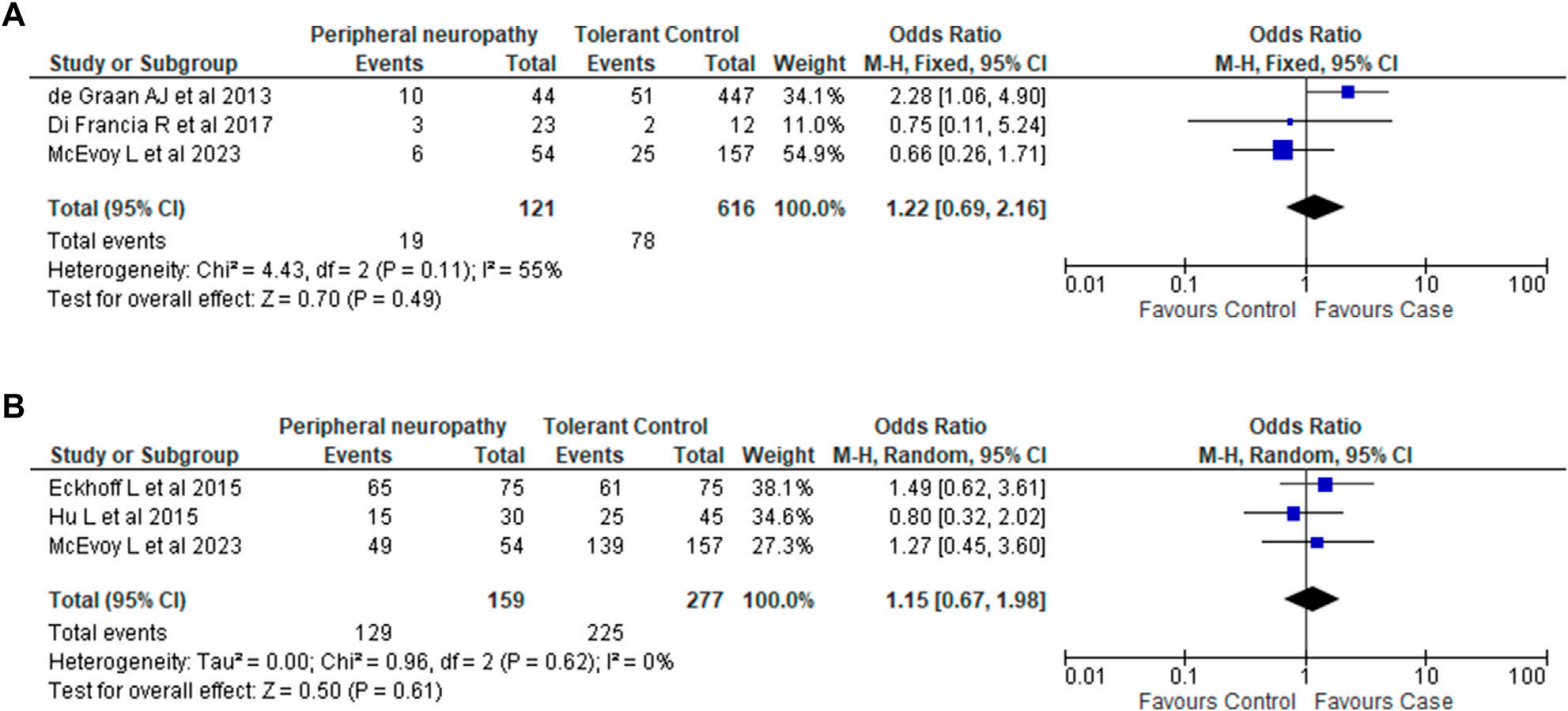
Association between CYP3A4*22 and CYP3A5*3 variants and taxane-induced peripheral neuropathy. **(A)**. Association between CYP3A4*22 and taxane-induced peripheral neuropathy. Analysis of *22 carriage (*1/*22 and *22/*22) vs. non-carriage (*1/*1). Note: The phenotype definition for cases in Di Francia et al. (2017) differed from our phenotype definition of Grade 2 PN and above. Di Francia et al. (2017) considered Grade 1 and above as cases. **(B)**. Association between CYP3A5*3 and taxane-induced peripheral neuropathy. Analysis of *3 homozygous carriage (*3/*3) vs. non-carriage and heterozygous carriage (*1/*1 and *1/*3).

The results of both Forest Plots are shown in Figure 2. Ethnicity information for all studies is described in Table 2. In our study, all participants were European Caucasians.

For CYP3A4*22, sufficient data was available from 2 studies (de Graan et al., 2013; Di Francia et al., 2017). Combining this with the data we generated showed that there was no association between CYP3A4*22 and PN (OR 1.22; 95% CI 0.69–2.16; *I* ^2^ 55%; *p* = 0.49).

For CYP3A5*3, sufficient data was available from 2 studies (Eckhoff et al., 2015a; Hu et al., 2016). Combining these two studies with the data from our candidate gene analysis again showed no association between CYP3A5*3 and PN (OR 1.15; 95% CI 0.67–1.98; *I* ^2^ = 0%; *p* = 0.61).

## 4 Discussion

Taxane chemotherapy is known to cause peripheral neuropathy, and is a leading cause of dose-reduction and/or premature treatment cessation, and has a detrimental impact on patients’ quality of life. Given the P450 enzymes involved in the biotransformation of taxanes, our study was designed to evaluate putative genetic associations between two candidate genes, CYP3A4*22 and CYP3A5*3, and development of TIPN. Both CYP3A4*22 and CYP3A5*3 are associated with decreased activity (Elens et al., 2011a; Elens et al., 2011b; Elens et al., 2011c; Elens et al., 2012; Elens et al., 2013a; Elens et al., 2013b; van der Weide and van der Weide, 2014; de Jonge et al., 2015; Sanchez Spitman et al., 2017; Mulder et al., 2021) and loss of function (Sanchez Spitman et al., 2017; Scheibner et al., 2018) respectively.

We undertook a systematic review, and also genotyped a cohort of patients treated with taxanes and oxaliplatin. In relation to *CYP3A4,* although de Graan *et al.* had identified an association between CYP3A4*22 carriers and PN (de Graan et al., 2013), other studies failed to demonstrate an association (Table 2). The results of our candidate gene study also failed to demonstrate an association between CYP3A4*22 and development of PN or PN severity (Table 4). The absence of subjects displaying a homozygous CYP3A4*22 genotype, however, is noteworthy. It is important to acknowledge that a potential risk associated with homozygous CYP3A4*22 genotypes cannot be completely ruled out due to our limited sample size. Further investigations with a larger, more diverse cohort, are warranted to comprehensively assess potential implications of homozygous CYP3A4*22 genotypes in TIPN. Similarly, an assessment of the current evidence failed to show a significant association between the CYP3A5*3 variant and development of PN or severity of PN (Bergmann et al., 2012; Bosó et al., 2014; Eckhoff et al., 2015a; Lambrechts et al., 2015; Schneider et al., 2015; Hu et al., 2016; Shen et al., 2023). Our candidate gene analysis also failed to demonstrate an association between CYP3A5*3 and development of PN or PN severity. For both variants, the lack of an association was confirmed by our meta-analyses.

Combined *CYP3A4* and *CYP3A5* genotypes were transformed into a CYP3A metaboliser phenotype classification with expected metabolic activities as previously defined (Sim et al., 2018). Carriers of CYP3A4*22 and CYP3A5*3 variant alleles have reduced metabolic activity and theoretically, would be at greater risk of toxicity. However, our data suggest that CYP3A metaboliser phenotype (*CYP3A4*/*CYP3A5*) is not a risk factor with a large effect size for taxane-induced peripheral neuropathy. However, we cannot exclude the possibility of a minor contribution, which would require a larger sample size.

We also studied a cohort of patients who had received oxaliplatin chemotherapy, which could be regarded as a separate “control” cohort. Oxaliplatin is not metabolised by CYP3A and therefore we did not expect to find any association with the *CYP3A* genetic polymorphisms. Indeed, this was borne out in our analysis with no association identified with either the individual alleles (Table 4) or the metaboliser status (Table 5).

Conventionally, paclitaxel is considered to be more frequently associated with peripheral neuropathy than docetaxel (Markman, 2003; Lee and Swain, 2006; Shimozuma et al., 2012; Kudlowitz and Muggia, 2013; Bhatnagar et al., 2014; Miltenburg and Boogerd, 2014). Our results support this. Our data suggest that development of peripheral neuropathy during chemotherapy is a greater risk in patients who have diabetes in keeping with previous literature (Hershman et al., 2016; Molassiotis et al., 2019). Advanced age has been a reported a risk factor in the literature (Tanabe et al., 2013; Schneider et al., 2015; Bao et al., 2016; Hershman et al., 2016; Tanabe et al., 2017; Bandos et al., 2018; Miaskowski et al., 2018; Molassiotis et al., 2019; Sánchez-Barroso et al., 2019; Hiramoto et al., 2022; Rattanakrong et al., 2022), and our data show a trend towards age being a risk factor, but this was not significant presumably because of a lack of power. Sex was also significantly associated with development of PN: 91% of cases were female.

The size of our candidate gene study is a limitation. A larger sample size may identify minor contributions from genetic variants predisposing individuals to increased susceptibility to TIPN, and allow for analysis of polygenic risk scores (PRS), rather than investigating individual alleles. Our study also lacks ethnic diversity: all participants were Caucasian. This is not rare. Genomic studies are commonly Euro-centric, with 97% of existing GWAS data from participants of European ancestry (Mills and Rahal, 2020; Pirmohamed, 2023). Our systematic review also showed an under-representation of non-European ancestry populations: 9 out of 12 studies were conducted in Europe, with an additional US study exclusively analysing patients genetically determined to be of European ancestry (Shen et al., 2023). However, evaluation of CYP3A4*22 and CYP3A5*3 allele frequencies in global populations (Supplementary Table S3) shows that the loss-of-function alleles are more common in European ancestry populations, and if an association exists with these variants and TIPN, it is more likely to be identified in this population. Concerning CYP3A5*3, there exists a significant challenge in establishing associations with TIPN in Caucasian populations due to the high frequency of the CYP3A5*3 variant. Further investigations in African ancestry populations, with a greater proportion of CYP3A5*1 alleles, may have greater power to detect differences in genetic predisposition to TIPN. Thus, future work should aim to improve ethnic diversity, serving to help address issues concerning heath disparities in genomic medicine and promote health equity. It would also identify novel loci predisposing to TIPN especially if genome-wide approaches are adopted.

In future studies, it will be important to consider not only the genetic factors, but also individual clinical factors with a view to generating multi-modal algorithms. For instance, multimorbidity, a recognised risk factor in CIPN, increases the prevalence of polypharmacy (Masnoon et al., 2017) and with that, the likelihood of drug- and gene-based interactions (Turner et al., 2020). With increasing cancer incidence and the increasing number of cancer survivors, together with changing population demographics, it is likely that the prevalence of CIPN will increase. It is therefore important that further research is conducted in this area, not only to identify predisposing factors, but also to develop biomarkers (Rossor and Reilly, 2022) which may allow earlier detection of toxicity, and offer insight into underlying mechanisms, pathogenesis and treatment (Rodwin et al., 2022).

It is also interesting to note that preventive strategies that can protect nerves from being damaged by taxane-based chemotherapy are being trialled (see Supplementary Table S4 which summarises trials registered on clinicaltrials.org). Some of these trials are investigating the use of neuroprotective agents, including vitamin E (Anoushirvani et al., 2018; Samuels and Ben-Arye, 2020; Chen et al., 2021) and acetyl-L-carnitine (Di Stefano et al., 2019), which have been shown to display some efficacy in preclinical studies. Alpha-lipoic acid has also been studied as an effective intervention for the treatment of diabetic neuropathy (Abubaker et al., 2022). Other studies are evaluating dose modifications (Sharma et al., 2020), and exercise programme (Kleckner et al., 2018; Müller et al., 2021; Chung et al., 2022) to prevent or manage PN. Despite some encouraging results, given that we do not fully understand the mechanisms of CIPN/TIPN, whether these intervention strategies will be successful is unclear, and continuing further research is needed.

Another factor which is important for future studies is to ensure that phenotypic assessment of patients is standardised. Standardization of phenotype definitions and consensus guidelines is an approach which has been employed with other ADRs (Aithal et al., 2011; Pirmohamed et al., 2011; Behr et al., 2012; Alfirevic et al., 2014; Carr et al., 2017; Nicoletti et al., 2021) as championed by the Phenotype Standardization Project (Pirmohamed et al., 2011). NCI-CTCAE is the most widely-used assessment tool for CIPN in clinical practice (Tan et al., 2019; Li et al., 2020; Selvy et al., 2021), yet despite this, a review identified 117 distinct CIPN assessment tools (McCrary et al., 2017). Heterogeneity in phenotype definitions, in terms of the particular assessment tool used, and the subjective nature of these tools may underestimate the prevalence and severity of CIPN (Cella et al., 2003; Cavaletti et al., 2010; Frigeni et al., 2011; Chow et al., 2012; Argyriou et al., 2014; Majithia et al., 2016; Beutler et al., 2017; Cliff et al., 2017; Le-Rademacher et al., 2017; Colvin, 2019; Kanda et al., 2019; Tan et al., 2019; Selvy et al., 2021). Thus, collaborative efforts to standardize detailed phenotype definitions and clinical assessment would help to mitigate diagnostic uncertainty, and therefore aid identification of genetic associations.

Currently, oncology is considered the most evolved field in personalised medicine. Despite this, use of PGx testing in taxane-based therapies is limited, in part due to speculation over positive impact on health outcomes, cost-effectiveness, and contradictory findings. A validated PGx panel assay for the prevention of neurotoxicity has been proposed (Di Francia et al., 2017), using pharmacogenomic profiles to stratify predicted treatment outcomes and optimize pharmacotherapy, but is not implemented, perhaps because of the contradictory genetic findings and lack of prospective evaluation. The importance of a robust study design in demonstrating the clinical utility of panel pharmacogenetic testing has been demonstrated recently in the PREPARE trial, where a 12-gene panel was able to reduce adverse drug reactions to a variety of compounds by 30% (Swen et al., 2023). Looking forward, as next-generation sequencing (NGS) technologies become more commonplace in research and clinical practice, the importance of low frequency variants (minor allele frequencies between 5% and 1%) and rare variants (minor allele frequency <1%) in drug response should be explored and evaluated using large-scale population studies supplemented by robust electronic health records or population biobanks with linked genomics data (Pirmohamed, 2023).

In conclusion, we did not demonstrate an association between TIPN and genetic polymorphisms in *CYP3A4* and *CYP3A5*. Our systematic review also shows some contradictory findings, but overall is consistent with our candidate gene analysis in failing to show an association, confirmed by the meta-analysis. Our study is limited by a small sample size, and so we cannot exclude a smaller effect size, and thus larger studies should be undertaken. However, in pharmacogenomics, this is not an easy task because of the need to identify patients treated with the same drug who have been accurately phenotyped. Given the narrow therapeutic index of many anti-cancer agents, and the wide range of toxicities which have been reported, this is an area which needs further study to improve the benefit-risk ratio. Although some successes in pharmacogenomics in relation to chemotherapy have been reported (Pirmohamed, 2023), much more work remains to be undertaken.

## Data Availability

The original contributions presented in the study are included in the article/Supplementary Material, further inquiries can be directed to the corresponding authors.

## Abbreviations

ADR, Adverse drug reaction; BMI, Body mass index; CIPN, Chemotherapy-induced peripheral neuropathy; CRF, Case report from; CYP, Cytochrome P450; DNA, Deoxyribonucleic acid; EM, Extensive metaboliser; GI, Gastrointestinal; GWAS, Genome wide association study; IM, Intermediate metaboliser; NCI-CTC AE, National Cancer Institute Common Terminology Criteria for Adverse Events; NHIR, National Institute for Health Research; PCR, Polymerase chain reaction; PGx, Pharmacogenomics; PIL, Patient information leaflet; PK, Pharmacokinetics; PM, Poor metaboliser; PN, Peripheral neuropathy; PRISMA, Preferred Reporting Items for Systematic Reviews and Meta-Analyses; PRS, Polygenic risk score; SNP, Single nucleotide polymorphism; TIPN, Taxane-induced peripheral neuropathy; WES, Whole-exome sequencing; WHO, World Health Organization.
