# Corrigendum: *CYP3A* genetic variation and taxane-induced peripheral neuropathy: a systematic review, meta-analysis, and candidate gene study

**DOI:** 10.3389/fphar.2023.1274075

**Published:** 2023-08-31

**Authors:** Laurence McEvoy, Joanne Cliff, Daniel F Carr, Andrea Jorgensen, Rosemary Lord, Munir Pirmohamed

**Affiliations:** ^1^ Department of Pharmacology and Therapeutics, University of Liverpool, Liverpool, United Kingdom; ^2^ Clatterbridge Cancer Centre, Liverpool, United Kingdom; ^3^ Health Data Science, University of Liverpool, Liverpool, United Kingdom

**Keywords:** chemotherapy, cytochrome P450, peripheral neuropathy, personalised medicine, pharmacogenetics

In the published article, there was an error in the legend and artwork for Figure 2 as published. Additional information relating to variant carriage or non-carriage needed. Forest Plot data was incorrectly reported. The corrected Figure 2 and its caption appears below.

**FIGURE 2:**
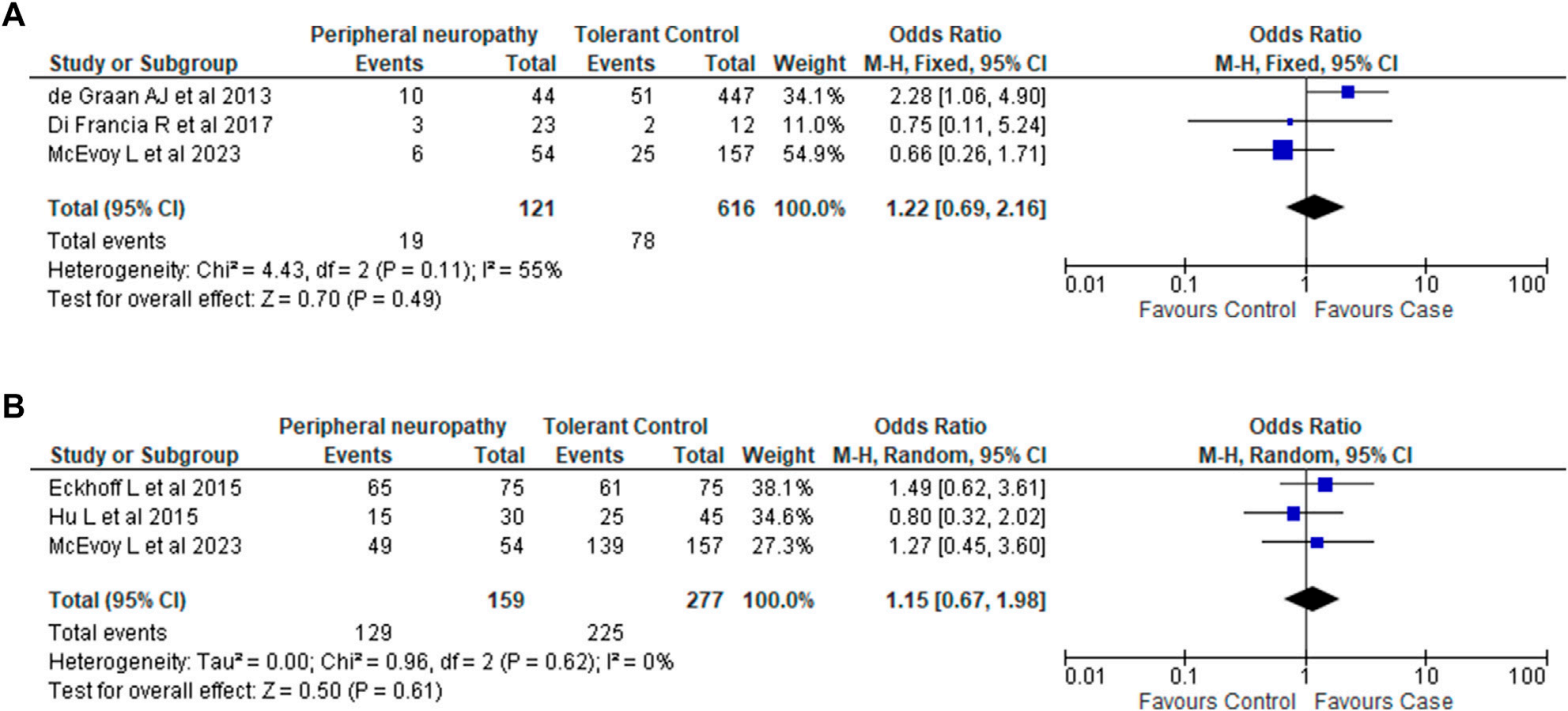
Association between CYP3A4*22 and CYP3A5*3 variants and taxane-induced peripheral neuropathy. **(A)**. Association between CYP3A4*22 and taxane-induced peripheral neuropathy. Analysis of *22 carriage (*1/*22 and *22/*22) vs. non-carriage (*1/*1). Note: The phenotype definition for cases in Di Francia *et al.* (2017) differed from our phenotype definition of Grade 2 PN and above. Di Francia et al. (2017) considered Grade 1 and above as cases. **(B)**. Association between CYP3A5*3 and taxane-induced peripheral neuropathy. Analysis of *3 homozygous carriage (*3/*3) vs. non-carriage and heterozygous carriage (*1/*1 and *1/*3).

In the published article, there was an error. Meta-analysis data from Figure 2 was incorrectly reported in the **Results**.

A correction has been made to **3 Results**, *3.4 Meta-analysis*, paragraphs 2 and 3. These sentences previously stated:

“For CYP3A4*22, sufficient data was available from 2 studies (de Graan et al., 2013; Di Francia et al., 2017). Combining this with the data we generated showed that there was no association between CYP3A4*22 and PN (OR 1.1; 95% CI 0.62-1.97; *I* ^*2*^ 42%; *p* = 0.74).

For CYP3A5*3, sufficient data was available from 2 studies (Eckhoff et al., 2015a; Hu et al., 2016). Combining these two studies with the data from our candidate gene analysis again showed no association between CYP3A5*3 and PN (OR 0.99; 95% CI 0.57-1.71; *I* ^*2*^ = 0%; *p* = 0.97).”

The corrected sentences appear below:

“For CYP3A4*22, sufficient data was available from 2 studies (**de Graan et al., 2013**; **Di Francia et al., 2017**). Combining this with the data we generated showed that there was no association between CYP3A4*22 and PN (OR 1.22; 95% CI 0.69–2.16; *I* ^2^ 55%; *p* = 0.49).

For CYP3A5*3, sufficient data was available from 2 studies (**Eckhoff et al., 2015a**; **Hu et al., 2016**). Combining these two studies with the data from our candidate gene analysis again showed no association between CYP3A5*3 and PN (OR 1.15; 95% CI 0.67–1.98; *I* ^2^ = 0%; *p* = 0.61).”

The authors apologize for this error and state that this does not change the scientific conclusions of the article in any way. The original article has been updated.

