# Supplementary material for: CYP3A genetic variation and taxane-induced peripheral neuropathy: a systematic review, meta-analysis, and candidate gene study
Source: Front Pharmacol. 2023 Jul 4;14:1178421. doi: 10.3389/fphar.2023.1178421 (PMC10352989; doi:10.3389/fphar.2023.1178421)
Supplement: Supplementary file 1 [file Table1.docx]

**SUPPLEMENTARY DATA**

***CYP3A* Genetic Variation and Taxane-Induced Peripheral Neuropathy: A Systematic Review, Meta-Analysis, and Candidate Gene Study**

**L. McEvoy^1^ |** **J. Cliff^2^** **|** **D. F. Carr ^1^| A. Jorgensen^3^ | R. Lord^2^ | M. Pirmohamed^1^**

**^1^ Department of Pharmacology and Therapeutics, University of Liverpool, Liverpool, UK; ^2^Clatterbridge Cancer Centre, Liverpool, UK; ^3^ Health Data Science, University of Liverpool, Liverpool, UK**

**Address:**

Wolfson Centre for Personalised Medicine,

Institute of Systems, Molecular and Integrative Biology (ISMIB)

University of Liverpool

Block A: Waterhouse Building

1-5 Brownlow Street

Liverpool    L69 3GL

**Email addresses:**

**Laurence McEvoy,** l.mcevoy@liverpool.ac.uk; **Joanne Cliff**, [joanne.cliff@nhs.net](mailto:joanne.cliff@nhs.net);
**Daniel F Carr,** dancarr@liverpool.ac.uk; **Andrea Jorgensen,** [aljorgen@liverpool.ac.uk](mailto:aljorgen@liverpool.ac.uk);
**Rosemary Lord**, [rosemarylord@nhs.net](mailto:rosemarylord@nhs.net); **Munir Pirmohamed,** munirp@liverpool.ac.uk

**KEY WORDS:** Chemotherapy; Cytochrome P450; Peripheral neuropathy; Personalised medicine; Pharmacogenetics.

**Table S1. Details of search strategy.**

|  | Search term |
| --- | --- |
| 1 | Chemotherapy |
| 2 | Docetaxel |
| 3 | Paclitaxel |
| 4 | Taxane |
| 5 | Geno* |
| 6 | Genetic* |
| 7 | Pharmacogen* |
| 8 | Haplotyp* |
| 9 | Variant |
| 10 | Allel* |
| 11 | SNP |
| 12 | Polymorphism |
| 13 | Neurotoxicity |
| 14 | Neuropath* |
| 15 | Peripheral Neuropathy |
| 16 | CYP3A4* |
| 17 | CYP3A5* |
| 18 | CYP3A |
| 19 | 1 OR 2 OR 3 OR 4 |
| 20 | 5 OR 6 OR 7 OR 8 OR 9 OR 10 OR 11 OR 12 |
| 21 | 13 OR 14 OR 15 |
| 22 | 16 OR 17 OR 18 |
| 23 | 19 AND 20 AND 21 AND 22 |
|  | * = any ending to the word |

**Table S2: Derivation of metaboliser status from the CYP3A genotypes.**

| Genotypes | CYP3A4*1/*1 | CYP3A4*1/*22 | CYP3A4*22/*22 |
| --- | --- | --- | --- |
| Taxane cohort | | | |
| CYP3A5*3/*3 | n = 158 IM (pm + em) | n = 30 PM (pm + im) | n = 0 PM (pm + pm) |
| CYP3A5*3/*1 | n = 22 EM (im + em) | n = 1 IM (im +im) | n = 0 PM (im + pm) |
| CYP3A5*1/*1 | n = 0 EM (em + em) | n = 0 EM (em + im) | n = 0 IM (em + pm) |
| Oxaliplatin cohort | | | |
| CYP3A5*3/*3 | n = 51 IM (pm + em) | n = 3 PM (pm + im) | n = 0 PM (pm + pm) |
| CYP3A5*3/*1 | n = 23 EM (im + em) | n = 0 IM (im +im) | n = 0 PM (im + pm) |
| CYP3A5*1/*1 | n = 0 EM (em + em) | n = 0 EM (em + im) | n = 0 IM (em + pm) |

**ABBREVIATIONS:** em, extensive metabolizer; im, intermediate metabolizer; pm, poor metabolizer

**Table S3. Global Allele Frequencies of *CYP3A4*22* and *CYP3A5*3* Variants Reported by the 1000Genomes Project.**

**A. *CYP3A4*22 (*rs35599367)**

| Population | Sample Size | Reference Allele | Alternative Allele |
| --- | --- | --- | --- |
| Global | 6404 | G = 0.9858 | A = 0.0142 |
| African | 1786 | G = 0.9989 | A = 0.0011 |
| Europe | 1266 | G = 0.9534 | A = 0.0466 |
| South Asian | 1202 | G = 0.9933 | A = 0.0067 |
| East Asian | 1170 | G = 1.0000 | A = 0.0000 |
| American | 980 | G = 0.978 | A = 0.022 |

**B*. CYP3A5*3 (*rs776746)**

| Population | Sample Size | Reference Allele | Alternative Allele |
| --- | --- | --- | --- |
| Global | 6404 | T = 0.3846 | C = 0.6154 |
| African | 1786 | T = 0.8175 | C = 0.1825 |
| Europe | 1266 | T = 0.0545 | C = 0.9455 |
| South Asian | 1202 | T = 0.3419 | C = 0.6581 |
| East Asian | 1170 | T = 0.2786 | C = 0.7214 |
| American | 980 | T = 0.201 | C = 0.799 |

**Table S4. Current Status of Clinical Trials in TIPN.**

| **Title** | **ClinicalTrials.gov identifier** | **Study Phase** | **Recruitment Status** | **Actual Study Start Date** | **Estimated Primary Completion Date** | **Study Type** | **Estimated Enrolment** | **Summary** |
| --- | --- | --- | --- | --- | --- | --- | --- | --- |
| Testing the Effects of Exercise on Chemotherapy-Induced Peripheral Neuropathy | NCT04888988 | Phase 2 | Recruiting | February 25, 2022 | February 1, 2025 | Interventional; Randomized Controlled Phase II Trial | 120 | This phase II trial studies whether using exercise is better than the usual approach for treating CIPN.  Assessment of CIPN: CIPN-20 total score. |
| Risk Prediction of Taxane Chemotherapy -Induced Peripheral Neuropathy (SENSE) | NCT04932031 |  | Recruiting | May 25, 2022 | November 30, 2024 | Observational; Prospective | 350 | Observational study to discover risk factors of CIPN in 350 patients with earlty stage breast cancer undergoing taxane-based chemotherapy.  Assessment of CIPN: CTCAE-CIPN and EORTC QLQ-CIPN20. |
| BXQ-350 Pharmacokinetic/Pharmacodynamic Study in Cancer Patients (RETRO) | NCT05291286 | Early Phase I | Recruiting | October 17, 2022 | March 2024 | Interventional; Randomized Early Phase I Trial | 20 | This study will assess pharmacokinetic (PK)/pharmacodynamic (PD) relationships and whether BXQ-350 may decrease the intensity and/or duration of CIPN, thereby improving quality of life.  Assessment of CIPN: CTCAE v5.0. |
| Biomarkers in Chemotherapy-Induced Peripheral Neuropathy: Better Tools and Understanding | NCT03348956 |  | Active, not recruiting | March1, 2018 | February 14, 2022 | Interventional; Pilot study | 30 | This plot study attempts to establish the feasibility of using tissue oxygen measurements and the protein, neurofilament light chain (NF-L), as potential biomarkers for CIPN. |
| International CIPN Assessment and Validation Study (ICAVS) | NCT04633655 |  | Recruiting | June 8, 2020 | May 1, 2023 | Observational; Prospective | 1000 | Observational study of CIPN patients, investigated prospectively, in order to assess responsiveness of a set of outcome measures in an international multi-centre study, involving various CIPN assessment methods. |

Data obtained from clinicaltrials.org
